# Sequential Brush Grafting for Chemically and Dimensionally Tolerant Directed Self-Assembly of Block Copolymers

**DOI:** 10.1021/acsami.2c16508

**Published:** 2022-12-19

**Authors:** Boyce S. Chang, Whitney S. Loo, Beihang Yu, Scott Dhuey, Lei Wan, Paul F. Nealey, Ricardo Ruiz

**Affiliations:** † Molecular Foundry, Lawrence Berkeley National Lab, Berkeley, California 94720, United States; ‡ Pritzker School of Molecular Engineering, University of Chicago, Chicago, Illinois 60637, United States; § Western Digital, San Jose, California 95119, United States; ∥ Materials Sciences Division, Argonne National Lab, Lemont, Illinois 60439, United States

**Keywords:** directed self-assembly, block copolymer, thin films, advanced lithography, defectivity

## Abstract

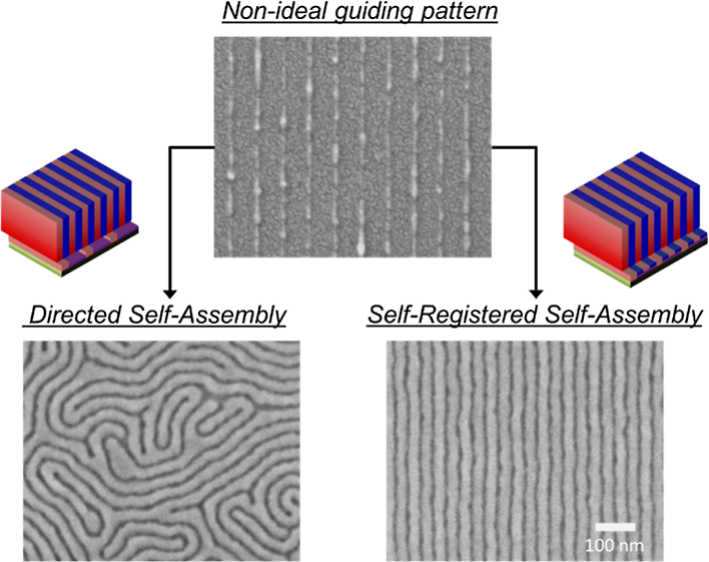

We report a method for the directed self-assembly (DSA) of block copolymers (BCPs) in which a first BCP film deploys homopolymer brushes, or “inks”, that sequentially graft onto the substrate’s surface *via* the interpenetration of polymer molecules during the thermal annealing of the polymer film on top of existing polymer brushes. By selecting polymer “inks” with the desired chemistry and appropriate relative molecular weights, it is possible to use brush interpenetration as a powerful technique to generate self-registered chemical contrast patterns at the same frequency as that of the domains of the BCP. The result is a process with a higher tolerance to dimensional and chemical imperfections in the guiding patterns, which we showcase by implementing DSA using homopolymer brushes for the guiding features as opposed to more robust cross-linkable mats. We find that the use of “inks” does not compromise the line width roughness, and the quality of the DSA as a lithographic mask is verified by implementing a robust “dry lift-off” pattern transfer.

## Introduction

Nearly 20 years ago, directed self-assembly (DSA) of block copolymers (BCPs) emerged as a promising alternative to reach previously inaccessible sub-lithographic dimensions with registration by combining top-down lithographic techniques with bottom-up self-assembly of BCP thin films.^1−3^ Achieving pattern perfection in DSA involves substantial interfacial energy engineering,^4,5^ a thorough understanding of the thermodynamics that drive the system toward equilibrium,^6−8^ and systematic control over the kinetic landscape to prevent the trapping of unwanted defects.^9,10^ In chemoepitaxial DSA, an array of “guiding” features alternating with “background” regions provide chemical contrast to direct the assembly of BCPs. The “guiding” features display a preferential wetting affinity for one of the two blocks in the BCP while the “background” region is tuned to be non-preferential for the unguided BCP domains.^4,11^ These chemical contrast patterns set the boundary conditions at the substrate interface, dictating much of the thermodynamic conditions critical for defect-free DSA.

End-grafted polymer brushes play a critical role in forming high-quality chemical contrast patterns. They were long introduced in chemoepitaxial DSA for their superior quality over self-assembled monolayers as surface modification layers capable of forming regions with well-defined chemical composition and wetting properties.^12−14^ However, they are susceptible to unwanted polymer interpenetration or brush insertion from other brushes or polymers applied during the fabrication process.^15^ To this end, cross-linkable mats were also introduced to guard against polymer interpenetration, preserving the surface chemical composition throughout the process.^11,14^ The most widely adopted “LiNe” process flow uses a cross-linkable polymer mat for the guiding feature alternating with OH-terminated random copolymer brushes having a composition that is carefully tuned according to the surface fraction of polymer blocks assembled in the background region.^4,11^ Pattern perfection was attained over the years by exerting utmost control over the chemical contrast patterns and carefully tuning the interfacial energy, chemical composition, and pattern quality of the guiding and background regions.^16−20^ Also, while BCPs are capable of realizing some form of pattern rectification,^21,22^ the tolerance window for correcting over defects and variations is bounded by a compromise between the line width of the guiding pattern (*W*_s_/*L*_0_),^4,6,16,23^ the commensurability and density of the guiding pitch (*L*_s_/*L*_0_),^4,11^ and the chemical composition of the background region:^4,6,14,24^ relaxing one parameter too much implies a more restrictive tolerance on the others. This points to some challenges ahead in extrapolating conventional DSA well below 10 nm where pattern variations and defects in the guiding features will be more common at the same time when the use of novel higher-χ materials,^25^ where χ is the Flory–Huggins interaction parameter between the polymer blocks of the BCP, will bring new challenges to fine tuning the background region composition. Ideally, a more powerful DSA approach would have more dynamic chemical contrast patterns capable of self-healing pattern imperfections and simultaneously self-tuning the chemical composition on the guiding patterns. This would concurrently relax the dimensions and the chemical composition specifications on the guiding patterns.

Recently, a new DSA workflow termed “self-registered self-assembly” (SRSA) that employed homopolymer brushes loaded inside a first, thin BCP film showed that the homopolymer brushes, referred to as inks, inserted themselves into the random brush background region, printing a new 1:1 chemical pattern comprising alternating polystyrene (PS)-preferential and poly(methyl methacrylate) (PMMA)-preferential domains.^26,27^ Upon rinsing, this denser chemical pattern can more robustly direct the assembly of a second, thicker BCP film without any defects. Previous work relied on cross-linkable PS (xPS) or PS-*r*-PMMA random mats in conjunction with the homopolymer inks,^28^ demonstrating a large density multiplication factor and an increased tolerance toward wider widths on the guiding stripes. Herein, we build on the SRSA concept to implement a DSA process that incrementally heals the quality and composition of the chemical contrast pattern by the sequential grafting of homopolymer brushes. We show that brush interpenetration could in fact be a desirable and powerful trait in widening the process window for a DSA process that is more tolerant toward dimensional imperfections and chemical composition mismatches on the pre-patterns.

In this work, we employ polymer brushes not only on the background region but also on the guiding features because brushes could be easier to scale below 10 nm than the cross-linkable polymer mats. We demonstrate a wide tolerance window employing only off-the-shelf polymer brushes, including sub-optimal composition random copolymers for the background region. We demonstrate that the process has a large tolerance over the quality of the guiding features and that the addition of the homopolymer inks does not compromise the line roughness of the final pattern. Finally, we test the quality of the assembly by implementing a robust “dry lift-off” protocol for pattern transfer based on atomic layer deposition akin to planarization methods.^29,30^ The results here should also make DSA a more accessible technique to research labs lacking direct access to custom-made polymer mats and brushes or without access to the latest e-beam or immersion lithography. While this work is still done with PS-*b*-PMMA, which will not extend to sub-10 nm,^31^ we anticipate that the self-healing properties on the guiding patterns and the wide process window margins developed here would be applicable to other higher-χ materials that could complement extreme ultraviolet (EUV) patterning.

## Materials and Methods

### Materials

Polymer brushes, BCPs, and homopolymers listed in Table 1 [PS, hydroxyl-terminated PS (S_OH_), PMMA (M_OH_), PS-PMMA (SrM_OH_) random copolymers, and PS-PMMA (SbM) BCPs] were obtained from a Polymer Source Inc. Toluene and chlorobenzene were obtained from Fisher Scientific, *N*-methylpyrrolidone (PG remover) was purchased from Kayaku Advanced Materials, Inc., and the tetramethylammonium hydroxide (TMAH)-based developer, MAD 533/S, was sourced from Micro Resist Technology. All chemicals were used as received. Si wafers were sourced from Addison Engineering.

**Table 1:** Description of the Polymers in This Study

name	symbol	MW_PS_ (kg mol^–1)^	MW_PMMA_ (kg mol^–1)^	*f*_PS_	PDI
**PS-OH**	**S**_**OH**_	**12.7**	—		**1.02**
**PMMA-OH**	**M**_**OH**_	—	**9.8**		**1.02**
**PS-*r*-PMMA-OH**	**SrM**_**OH**_	**2.2**	**2.9**	**0.43**	**1.45**
**PS-*b*-PMMA**	**SbM**	**25**	**26**	**0.50**	**1.03**
**PS**	**PS**	**30**	—		**1.10**

### Polymer Film and Substrate Preparation

All polymers used are dissolved in toluene at 1 wt % and filtered through 0.45 and 0.02 μm syringe filters. Si wafers are pre-treated with UV–ozone for 5 min. Films are spin-coated at 1000–5000 rpm depending on the target thickness.

### Preparation of Polymer-Grafted Substrates and Brush Exchange Experiments

A solution containing brush molecules is spin-coated on a Si wafer and subsequently annealed at 260 °C for 15 min. The wafer is then sonicated in NMP for 5 min 3 times to remove the unreacted polymer chains. Sequential brush insertion experiments are performed by repeating the spin coating, annealing, and rinsing steps on existing brush-coated wafers.

### Directed Self-Assembly

In a typical experiment, PMMA (950 kDa) is diluted to 1% in chlorobenzene and spin-coated onto a Si wafer grafted with a monolayer of S_OH_ at 3000 rpm to give a thickness of 45 nm and serve as the resist for electron beam lithography. The line/space patterns are then exposed on a Raith EBPG 5200 electron beam lithography system at 100 kV and a 2 nA beam current. The PMMA is then developed using a high contrast cold development process consisting of 7:3 IPA/water at 5 °C ultrasonicated for 100 s. This resulted in arrays of lines with a 64 nm (2*L*_0_) pitch with various line widths between 10 and 22 nm (0.3*L*_0_–0.7*L*_0_). The wafer is then dry-etched using O_2_ plasma to remove the exposed PS brush. The remaining resist is removed through three rounds of sonication in NMP for 5 min, followed by one round of sonication in chlorobenzene for 5 min. A 43 mol % styrene SrM_OH_ brush is used to backfill the exposed Si according to the above instructions. The SbM BCP (20–40 nm) is then spin-coated and annealed at 260 °C for 15 min.

### Dry Lift-Off Pattern Transfer

PMMA is selectively etched using O_2_ plasma from the BCP film, with an estimated 10% over-etch to ensure complete removal. 10 nm of the Al_2_O_3_ conformal coating was deposited using thermal atomic layer deposition (ALD) at 40 °C. This planarization layer was etched with BCl_3_ plasma to expose the interdigitated PS/Al_2_O_3_ stripes. The PS pattern is subsequently removed via dry etching with O_2_ plasma. The Si substrate is etched with CF_4_/CHF_3_/Ar plasma, and the remaining Al_2_O_3_ was removed using TMAH wet etch with the MAD533/S developer.

### Characterization

Scanning electron micrographs are obtained using a Zeiss Ultra 60. Atomic force microscopy is performed using a Bruker Dimension Icon. Water contact angle measurements are taken with a Kruss DSA100E. Surface infrared spectroscopy measurements are made using a Thermo-Fischer Nicolet iS50 FTIR equipped with variable angle reflectance accessory by Harrick VariGATR and a germanium crystal. The film thickness is measured with ellipsometry with a JA Woollam M-20000 DI ellipsometer.

## Results and Discussion

**Figure 1:**
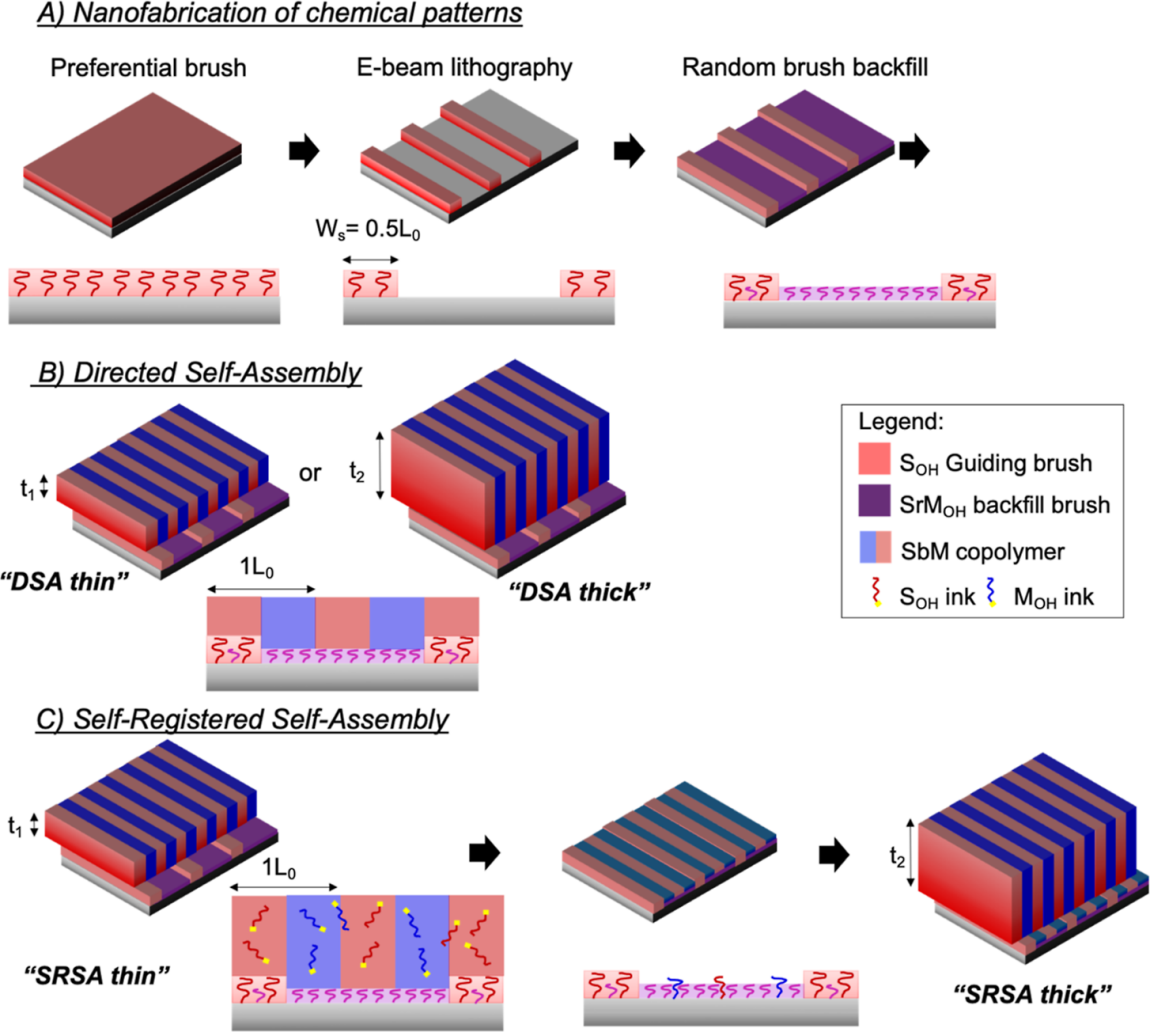
Process flow for the (a) nanofabrication of the chemical guiding patterns, (b) workflow 1: DSA, and (c) workflow 2: SRSA.

Table 1 describes the polymers used in this work, their molecular weights, and the symbols used throughout this paper. Figure 1a shows a schematic workflow for the fabrication of *all-brush* chemical contrast patterns. First, a PS polymer brush, S_OH_, with chemical affinity toward the S block of the PS-*b*-PMMA copolymer, is grafted on a Si substrate and covered by a layer of e-beam resist followed by electron beam lithography (EBL) to generate periodic lines (2*L*_0_ apart) on the resist that serve as a soft mask as described in the Materials and Methods Section. Subsequently, an O_2_ plasma etch clears off the exposed S_OH_. The remaining e-beam resist is stripped in NMP, and the background region is backfilled by grafting a shorter random copolymer brush composed of PS-*r*-PMMA with 43 mol % styrene (SrM_OH_). Figure 1b shows a variation of the LiNe workflow for DSA with the exception that the guiding features are made of an S_OH_ brush instead of a cross-linkable PS mat. Upon spin-coating and thermal annealing at 260 °C, the PS-b-PMMA copolymer with a full pitch of *L*_0_ = 32 nm forms lamellar films perpendicularly oriented to the substrate, which are guided by the chemical patterns. Alignment of the lamellae is achieved as the S_OH_ guiding brush anchors every other S domain on the SbM film, whereas the SrM_OH_ random brush remains non-selective and permits wetting from both S and M domains. The success of this process highlights the ability of BCP DSA to multiply the number of features and shrink pitch sizes obtained from the EBL.^32^Figure 1c shows an SRSA workflow, an alternative that utilizes the same chemical patterns shown in Figure 1a, but here, additional homopolymer brushes (S_OH_ and M_OH_) are blended into the SbM solution (5 wt % each) prior to spin-coating. We refer to the homopolymer brushes as “inks”. Both S_OH_ and M_OH_ are chosen such that their chains are longer than the SrM_OH_ chains in the background region, but with M_OH_ having shorter chains than the S_OH_ chains in the guiding patterns. Upon thermal annealing, the inks phase-separate into the S and M domains of the SbM film, and a portion of these inks interpenetrate the SrM_OH_ layer and graft onto the substrate, becoming part of the brush monolayer. The grafted inks, having longer chains than that of the SrM_OH_, stand above the background region, registering the individual BCP domains into chemical patterns on the Si substrate. Subsequently, the ink-embedded SbM film is washed away, revealing a self-registered 1:1 guiding pattern. Next, a thicker (*t* > 1*L*_0_), second SbM film is spin-coated and annealed on to the patterned substrate (Figure 1c). Previous work on SRSA^26^ has shown that the process window with regard to the guiding line width (*W*_s_) for defect-free DSA widely expanded compared to conventional DSA. In ref (26), the guiding features were still made by cross-linkable mats, and the backfilling brush was a highly optimized, custom-made random brush. Our work expands beyond that in ref (26) by using off-the-shelf polymer brushes as the guiding pattern and a random backfill brush with a sub-optimal polymer composition for the given density multiplication. According to theoretical calculations, the ideal composition for the backfill neutral brush is 35 mol % styrene for a 2× density multiplication, whereas we are able to achieve high-quality DSA with a 43 mol % styrene PS-*r*-PMMA random brush for a variety of guide stripe widths.^4^

**Figure 2:**
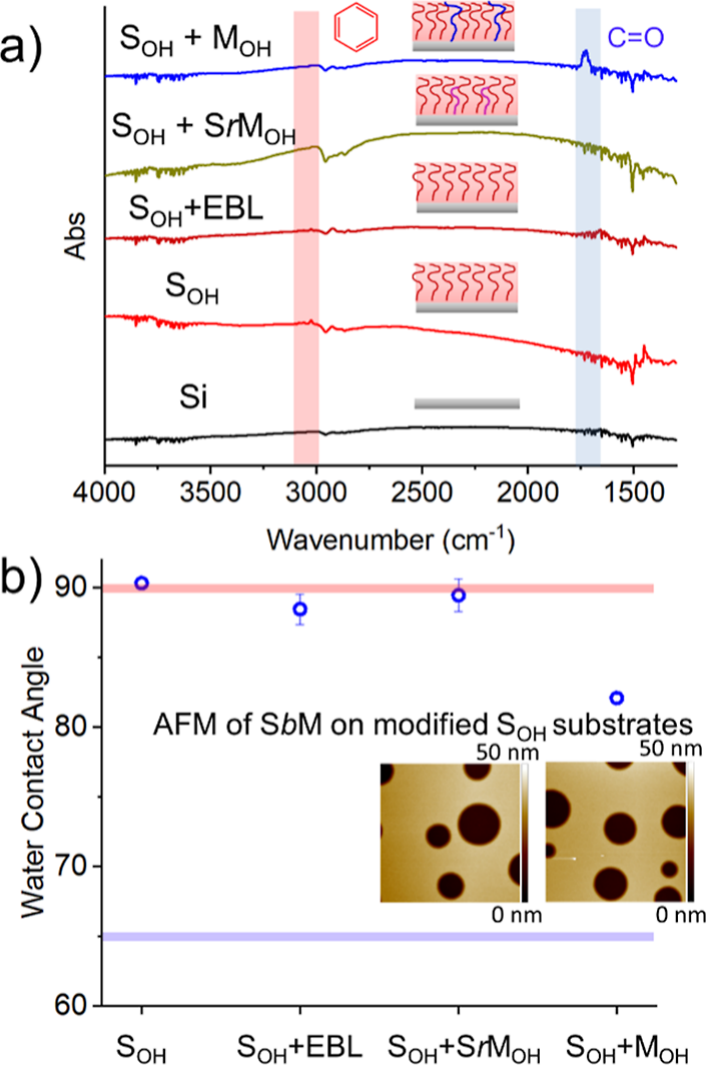
Retention of surface properties and composition properties of the guide stripes. (A) Absorbance from FTIR measurements of pristine Si (black), S_OH_ (red), S_OH_ + SrM (gold), and S_OH_ + M_OH_ (blue). The wavenumber region highlighted in red shows the PS signature (aromatic C–H) and that in blue shows the PMMA signature (C=O). After E-beam patterning, the S_OH_ substrate does not show any signatures of PMMA contamination (see the Supporting Information). (B) Water contact angle of the S_OH_ substrate throughout the DSA process: S_OH_, S_OH_ + Ebeam, S_OH_ + SrM, and S_OH_ + M_OH_. The water contact angle of S_OH_ only changes when the pure M_OH_ brush is grafted. The insets show the AFM images (scan size: 5 μm × 5 μm) after annealing of a 1.75 *L*_0_ thick SbM film on top of the modified S_OH_ substrates and show hole features indicating a preferential substrate for PS.

The use of an all-brush chemical pattern for DSA requires retention of the wetting properties of the guiding brush, S_OH_, over lithographic processes. Shifts in chemical affinity of random copolymer neutral brushes have been reported after chemical processing from EBL including deposition of the resist, electron beam exposure, and pattern development.^33^ Furthermore, the backfilling random brush step in Figure 1a requires that S_OH_ retains its guiding ability after grafting SrM_OH_. Evidence suggests that chain insertions^15^ or exchange^26,34^ could occur when a polymer monolayer is thermally annealed in the presence of polymer chains that possess binding groups such as −OH. To mitigate this effect, a lower-molecular-weight SrM_OH_ brush is selected relative to S_OH_ (Table 1) such that the surface affinity of the guiding stripes is retained even if a fraction of SrM_OH_ inserts itself in the S_OH_ stripes. We applied surface FTIR (Figure 2a) and water contact angle (Figure 2b) measurements at each step of the DSA workflow in control samples on un-patterned Si substrates. We denote the sequential deposition of polymer brush layers with a “+” such that the term “S_OH_ + SrM_OH_” relates to a S_OH_ monolayer substrate that has been subsequently modified through the sequential addition of a SrM_OH_ layer, thermally annealed at 260 °C, and then rinsed to remove any ungrafted polymer, revealing a new monolayer of the polymer brush. The carbonyl C=O (∼1750 cm^–1^) stretch was used as a proxy for PMMA in FTIR and is highlighted in blue, while the aromatic C–H stretch (∼3200 cm^–1^) was used as a proxy for PS and is highlighted in red. Similarly, the bulk water contact angles for PMMA and PS are highlighted in blue and red, respectively, in Figure 2b. Both FTIR and water contact angle measurements demonstrate that thermal annealing of S_OH_ in the presence of SrM_OH_ does not significantly affect the chemical structure or wetting properties of the initial S_OH_ brush layer. Only thermal annealing in the presence of a pure M_OH_ brush changes the chemistry and water contact angle of the S_OH_ substrate as shown by the appearance of a carbonyl peak near 1750 cm^–1^ in the FTIR spectra and a lower water contact angle. However, the water contact angle only drops to 80°, which is still significantly higher than that of pure PMMA (65°), indicating that there was no full exchange between S_OH_ and M_OH_ and that the surface energy of the S_OH_ + M_OH_ substrate is closer to that of bulk PS than bulk PMMA. Furthermore, the application of an e-beam resist layer and subsequent EBL processing (in areas not exposed to the electron beam) did not alter the wetting of S_OH_ (S_OH_ + EBL in Figure 2). We performed hole-island tests, wherein an SbM film with a thickness of 1.75*L*_0_ is deposited on both S_OH_ + SrM_OH_ and S_OH_ + M_OH_ substrates to probe the surface chemical affinity of the substrate (inset Figure 2b). Under these conditions, both films showed 1*L*_0_ hole features indicative of a PS-preferential substrate, confirming the preserved chemical affinity and wetting properties of the guiding S_OH_ brush.^5^ Therefore, despite the decrease in water contact angle and the presence of PMMA chemistry within the S_OH_ + M_OH_ substrate, there is no sufficient addition of M_OH_ nor a significant removal of S_OH_ chains to change the chemical affinity of the S_OH_ substrate. Thus, the S_OH_ guide stripes retain their chemical affinity for S after grafting of SrM_OH_ and M_OH_ during the backfilling and SRSA processes, respectively.

In previous studies where xPS mats were used as the guiding material, a six degree drop in the water contact angle was observed after lithographic patterning, and an additional two degree drop was observed after grafting of the neutral brush, resulting in a total decrease from 91 to 82.5°.^6^ These results suggest that S_OH_ guiding patterns retain their hydrophobicity and PS wetting properties similar to or perhaps even better than the conventional xPS mats. In addition, there have been previous reports of unavoidable non-preferential brush grafting on top of the xPS substrate, which alters the wetting properties of the guiding stripes and complicates efforts to control the pattern surface energy.^35^ Furthermore, polymer brushes are extremely tunable in both film thickness, by controlling the polymer molecular weight, and wetting properties, by tuning the brush chemistry. As the community scales down DSA toward smaller dimensions, the thickness and width of the guiding stripes will need to be scaled down accordingly;^26^ we hypothesize that polymer brush substrates may be able to form uniform films at smaller thickness values than cross-linkable polymer mats, which have previously shown uniformity challenges below 8 nm thickness.^16,36^

**Figure 3:**
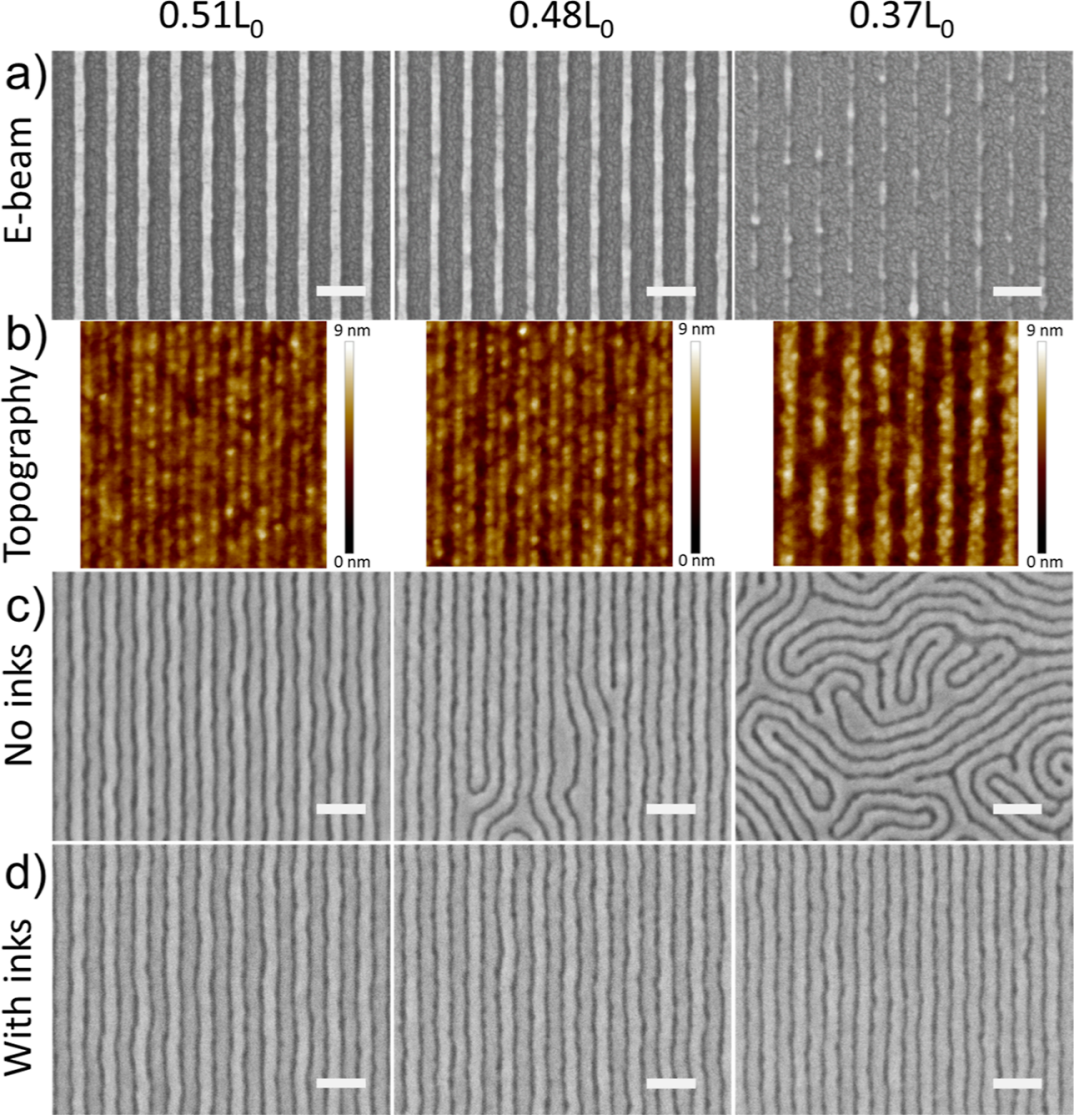
Effect of the PS brush guide stripe width on the DSA quality. (A) E-beam pattern sputtered with 1.5 nm of Au/Pd following a dry etch. (B) Topography of the PS brush guide stripes from AFM measurements after removal of the PMMA resist. (C) DSA of a thick film (1.25 *L*_0_ = 40 nm) on each E-beam pattern without homopolymer inks (DSA Workflow, Figure 1b). (D) DSA of a thick film (1.25 *L*_0_ 40 nm) on each E-beam pattern with homopolymer inks (SRSA Workflow, Figure 1c). Scale bar = 100 nm.

Demonstrating the robustness of the polymer brush, we proceeded with the complete workflow for DSA beginning from the EBL patterns, stripping of the resist, backfilling with the SrM_OH_ brush, and finally depositing the SbM film (Figure 3). The doses of electron beam exposure were varied to obtain line patterns of different widths, *W*_s_, between 0.37*L*_0_ and 0.51*L*_0_ (Figure 3a). Relaxing the guiding line to *W*_s_ > 0.5*L*_0_ is beneficial only with density multiplication factors that are larger than 2×; the effect of wider guide stripes on the SRSA alignment for 3× and 5× density multiplication has been reported in ref (26). At the highest doses, the patterns were over-dosed, leading to poor structural integrity of the unetched resist as well as the underlying brush; broken resist lines after etching are a signature of over-dosed patterns as seen in the right column of Figure 3a,b. Furthermore, as a result of O_2_ plasma etching, sidewall deposition (∼1 nm) of etched materials can be observed in the topography map of the S_OH_ guiding patterns *via* AFM. The feature of the sidewall appears thicker than expected in AFM (Figure 3b) than in SEM (Figure 3a) likely due to the differences in topography: the patterns shown in Figure 3a include the EBL resist and 1.5 nm of Au/Pd alloy on top of the guide stripes (total thickness of ∼25 nm), while those in Figure 3b highlight the topography of the pristine S_OH_ guide stripes (total thickness of ∼5 nm). In addition, the S_OH_ guiding patterns do not exhibit the footing, or widening at the substrate, that is characteristic of xPS guiding patterns and has been known to induce topography-related defects in the resulting polymer pattern.^23,37^ The importance of the pattern geometry on the quality of DSA was demonstrated for xPS mats where line widths between 0.5 and 0.7*L*_0_ afforded the perfect DSA in lamellar BCPs.^11^ Similarly, for SbM films with *t* > 1*L*_0_, we observe the perfect DSA only when *W*_s_ ∼ 0.5*L*_0_. At smaller values of *W*_s_, defects begin to appear and finally, for the damaged brush patterns with *W*_s_ = 0.37*L*_0_, fingerprint features were observed, indicating no guiding from the underlying patterns (Figure 3c). However, when the SRSA procedure is applied where a 1:1 guiding of the BCP is expected to occur, all three patterns showed a perfect, defect-free DSA (Figure 3d). This result highlights the ability of subsequent grafting of polymer brushes embedded in the SbM matrix to (i) improve the chemical contrast of the patterns and (ii) self-correct imperfect features produced by EBL, thus widening the process window for long-range DSA.

While the workflow including DSA and SRSA provided the desired outcome, the mechanism behind the improved process window remains elusive because of challenges in imaging the 1:1 registered pattern from SRSA. AFM micrographs of the brush layer were taken throughout the workflow in an attempt to capture the evolution of the chemical contrast patterns. Supporting Information Figure S1a shows the initial S_OH_ guiding stripes. In S1b, the addition of the SrM_OH_ is easy to deduce from the reduced step height between the guiding line and the background region, but after the use of inks in SRSA in Figure S1c, the background region does not show any topographic features from the anticipated interpenetration of S_OH_ and M_OH_ inks, making it difficult to assess the extent of any brush interpenetration. Despite observing slight changes to the side wall profile, we would like to emphasize that their formation is not controlled as it is a byproduct of plasma etching (Supporting Information Figure S1). This is in stark contrast to “molecular transfer printing” developed by Nealey and co-workers, where embedded polymer brushes were grafted onto pristine Si substrates to generate a new “daughter” chemical pattern to be used for subsequent rounds of DSA.^28^ In that work, the polymer brushes were observable in both SEM and AFM albeit the BCP feature sizes were ∼2× larger compared to this work. On the other hand, SRSA requires the embedded chains to graft onto the chemically patterned substrate, meaning an insertion into a vacant site or exchange of polymer brushes. These processes necessitate diffusion of the polymer chains across the initial brush layer, thus potentially compromising grafting efficiency and speed. In addition, the chemical and topographical contrast between the grafted neutral brush and any additional homopolymer inks could be extremely limited.

**Figure 4:**
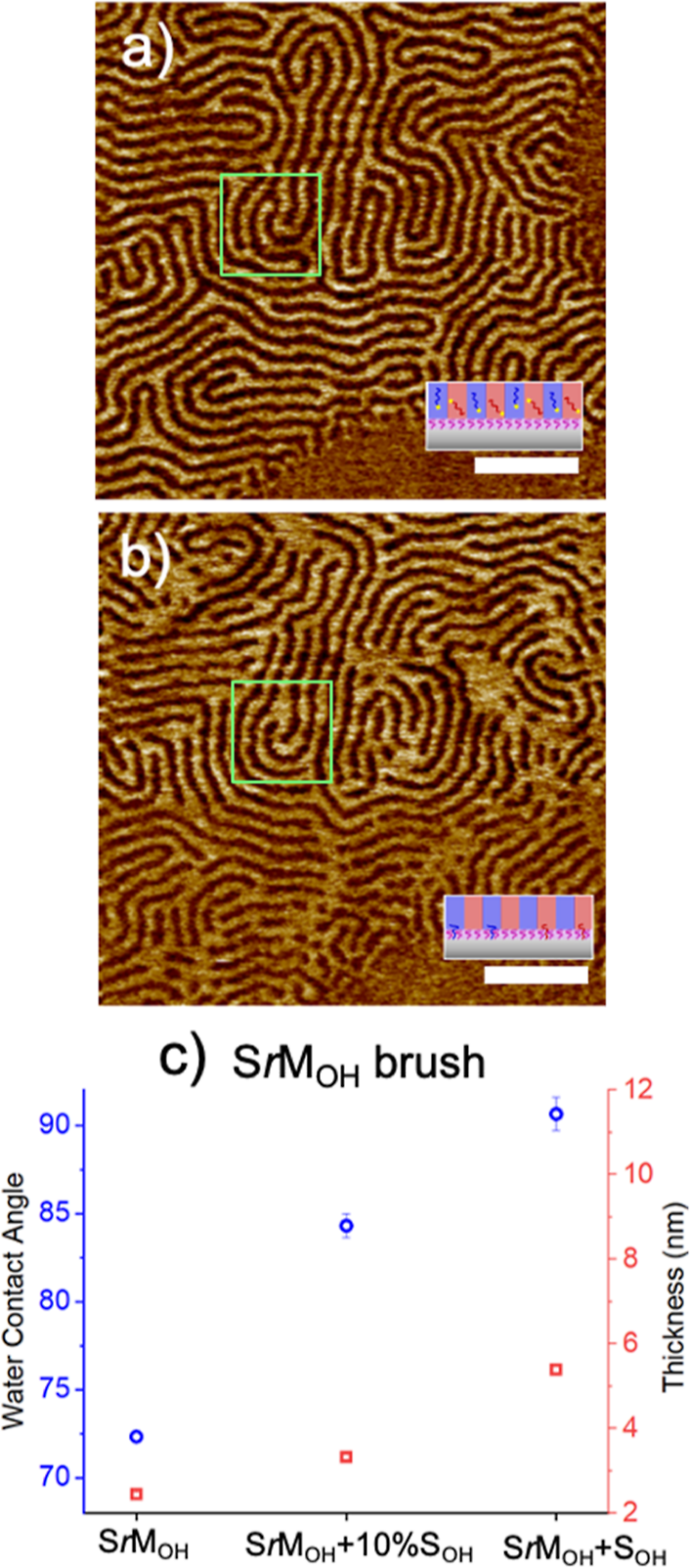
DSA Mechanism (1:1 Guiding patterns) (a) fingerprint formed from the initial SRSA film [S*r*M_OH_ + S*b*M(20)/S_OH_(1)/M_OH_(1)]. (b) Subsequent SbM film deposited on the registered substrate after removing the SRSA film [S*r*M_OH_(S_OH_,M_OH_)+S*b*M]. Scale bar = 200 nm. The green squares highlight equivalent regions in both images. (c) S*r*M_OH_ brush substituted with films containing different concentrations of S_OH_, SrM_OH_ + 10% S_OH_, and SrM_OH_ + S_OH_. Brush layer transitions toward S_OH_ in both water contact angle (blue, left *y*-axis) and thickness as measured by ellipsometry (red, right *y*-axis). Error bars refer to standard deviation, *n* = 3.

Therefore, the question remains as to how deterministic the new 1:1 patterns are or how strong the new chemical contrast is. In an attempt to answer this question, we tracked SRSA SbM fingerprint patterns containing embedded polymer inks spun-coated on a neutral brush, S*r*M_OH_ + S*b*M(20)/S_OH_(1)/M_OH_(1), where the numbers in parentheses correlate to the relative volume fraction of each component in the solution for a total concentration of 1 wt % (Figure 4a). Using a fingerprint pattern instead of DSA allows us to isolate the effect of the guiding brush during SRSA. Following the removal of the polymer film, a fresh SbM film is spin-coated on the SRSA registered substrate in order to track the fingerprints formed in the same location (Figure 4b). Low curvature features were successfully reconstructed in the new film, validating that 1:1 guiding occurs from the inserted brushes in SRSA. However, features with a higher curvature, see the highlighted green square in Figure 4, were noticeably poorly translated, which could be a consequence of the pattern at the substrate not matching what was observed at the surface or the inks not diffusing equally well on the highly curved portions of the pattern as suggested by ref (28). We also measured the change in water contact angle and thickness of the SrM_OH_ brush layer after modification by SRSA, by embedding 10 wt % S_OH_ inks into a PS homopolymer film, thus, simulating SRSA within a single domain of the BCP, shown in Figure 4c. After thermal annealing, the contact angle increased from ∼73 to ∼85°, indicating the successful grafting of S_OH_ and change in chemical affinity of the brush layer. Surprisingly, the thickness of the film increased marginally from 2.7 to 3.1 nm, which implies that only a limited number of S_OH_ chains are grafted onto the SrM_OH_ substrate. This is validated when a pure S_OH_ film is deposited and thermally annealed over SrM_OH_; the thickness of the film increases nearly two-fold (5.2 nm) and contact angle by ∼90°, matching that of a pure S_OH_ brush layer.

**Figure 5:**
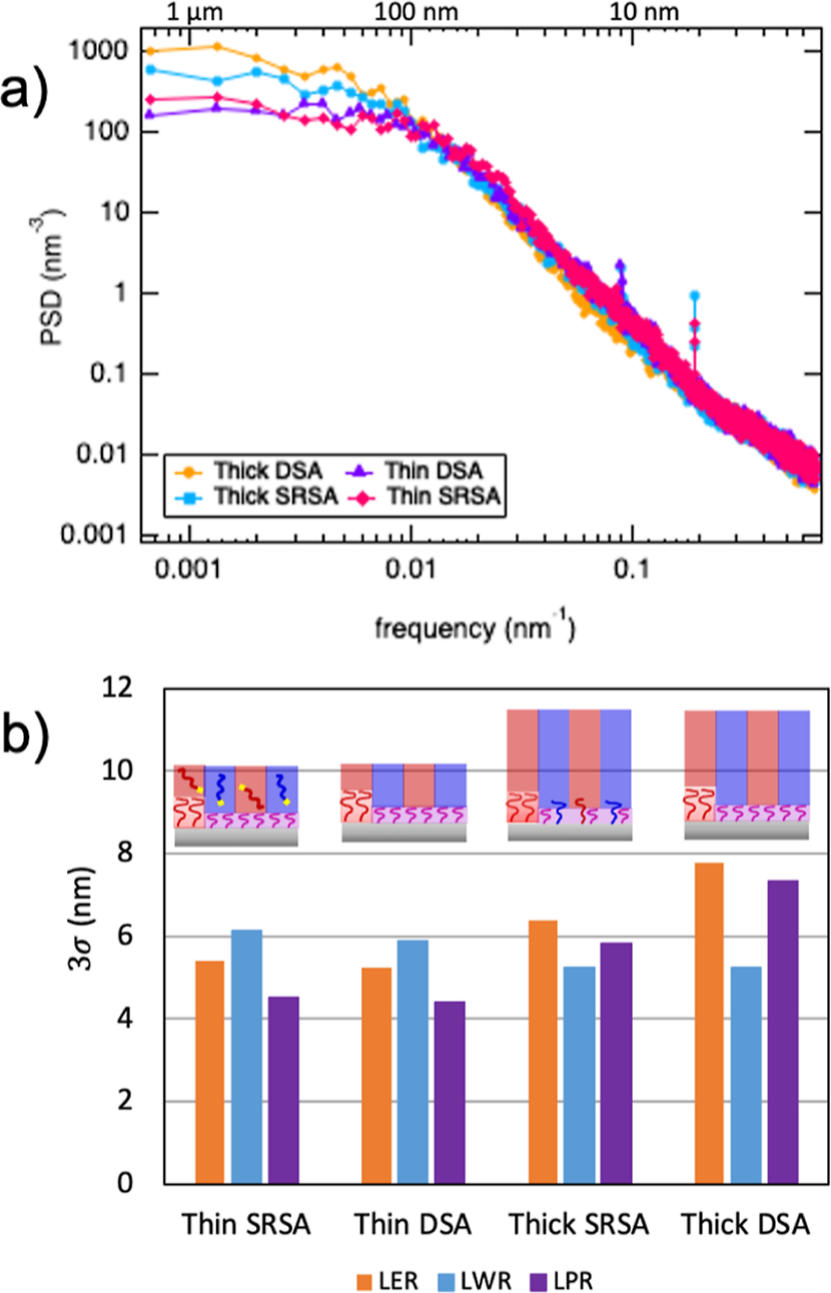
PS roughness calculations for BCP patterns after different DSA protocols. (A) Power spectral density of LPR for the PS domains for the various DSA protocols: thin and thick films after DSA (no inks) and SRSA (with inks). (B) Line roughness quantified by 3σ of the power spectral density of the PS domain from the various DSA processing conditions showing the LER, LWR, and LPR.

BCP line roughness is an important performance metric for applying DSA to semiconductor manufacturing and lithography,^38,39^ and therefore, we quantified the effect of the introduction of the homopolymer inks on the line roughness. Figure 5a shows an example power spectral density (PSD) profile of the PS line placement roughness (LPR) for the various DSA conditions: DSA versus SRSA and thin versus thick films, which are shown schematically in Figure 1. The PSD profile was calculated according to the imec protocol outlined in ref (38). The top *y*-axis shows the real-space dimensions that correspond to the frequency, shown as the bottom *y*-axis. The PS line placement roughness quantifies the fluctuations of the centroid, or position, of the PS domain at different length-scales described in the frequency space.^40^ At low frequencies (below 0.02 nm^–1^, which corresponds to length-scales above 50 nm), the placement fluctuations plateau as a direct result of the guiding properties of the underlying pattern (Figure 5a). The magnitudes of the placement fluctuations at low frequencies of the thick films for both DSA and SRSA are higher than that of the thin films, as expected. The guiding influence of the substrate diminishes as the film thickness increases, resulting in weaker guiding at the surface of the thicker films and therefore higher fluctuations in domain placement. At intermediate and high frequencies (above 0.02 nm^–1^, which corresponds to length-scales below 50 nm), the PSDs lie on top of one another, indicating that at length-scales smaller than the pitch of the BCP, there are no differences in placement fluctuations between the SbM polymer films. The remaining PSD profiles for the line edge, line width, and line placement roughness are provided in the Supporting Information (Figure S2). Figure 5b shows the effects of DSA processing conditions on the overall roughness, 3σ, calculated from the PSD profiles of the line edge roughness (LER), line width roughness (LWR), and LPR of the PS lines for the SbM films. The overall roughness, 3σ, for the PMMA lines as well as the correlation coefficients for the PS and PMMA lines are provided in the Supporting Information (Figures S3 and S4). Our values are similar to those calculated by simulations of an analogous PS-*b*-PMMA copolymer.^41^ Similar to the LPR, the LER of the thick films for both DSA and SRSA is larger than that of the corresponding thin films. The LWR for the PS lines is fairly constant for the various DSA processing conditions. The SRSA thin films have a larger LWR when compared with the DSA thin film and both thick films. This is likely induced by the homopolymer inks loaded in the SRSA thin film. While some homopolymer inks have grafted to the substrate, we hypothesize that many brush molecules remain in the BCP film. We believe that this increase in the polydispersity of the BCP film, and therefore the local fluctuations lead to the increase in LWR.^42^ This is consistent with previous studies where an asymmetrical distribution of the free polymer within a domain and between neighboring domains has been shown to increase the LWR.^43^ Following rinsing of the ink-containing polymer film and subsequent annealing of a thick SbM film on top of the registered substrate, the PS line width roughness returns to the value seen in the DSA films. Therefore, the registered inks grafted to the chemically patterned substrate do not increase the final line width roughness of the copolymer film. In addition, the line edge and PS line placement roughness of the SRSA thick film are consistently lower than those of the DSA film. The differences come from the low-frequency components for wavelengths larger than ∼100 nm, indicating that the denser 1:1 guiding from the registered inks decreases the amplitude of line fluctuations at low frequencies compared to the more relaxed 2:1 guiding in the original DSA pattern. Therefore, the benefits that come with the more tolerant SRSA process come without inducing detrimental effects to the pattern roughness.

**Figure 6:**
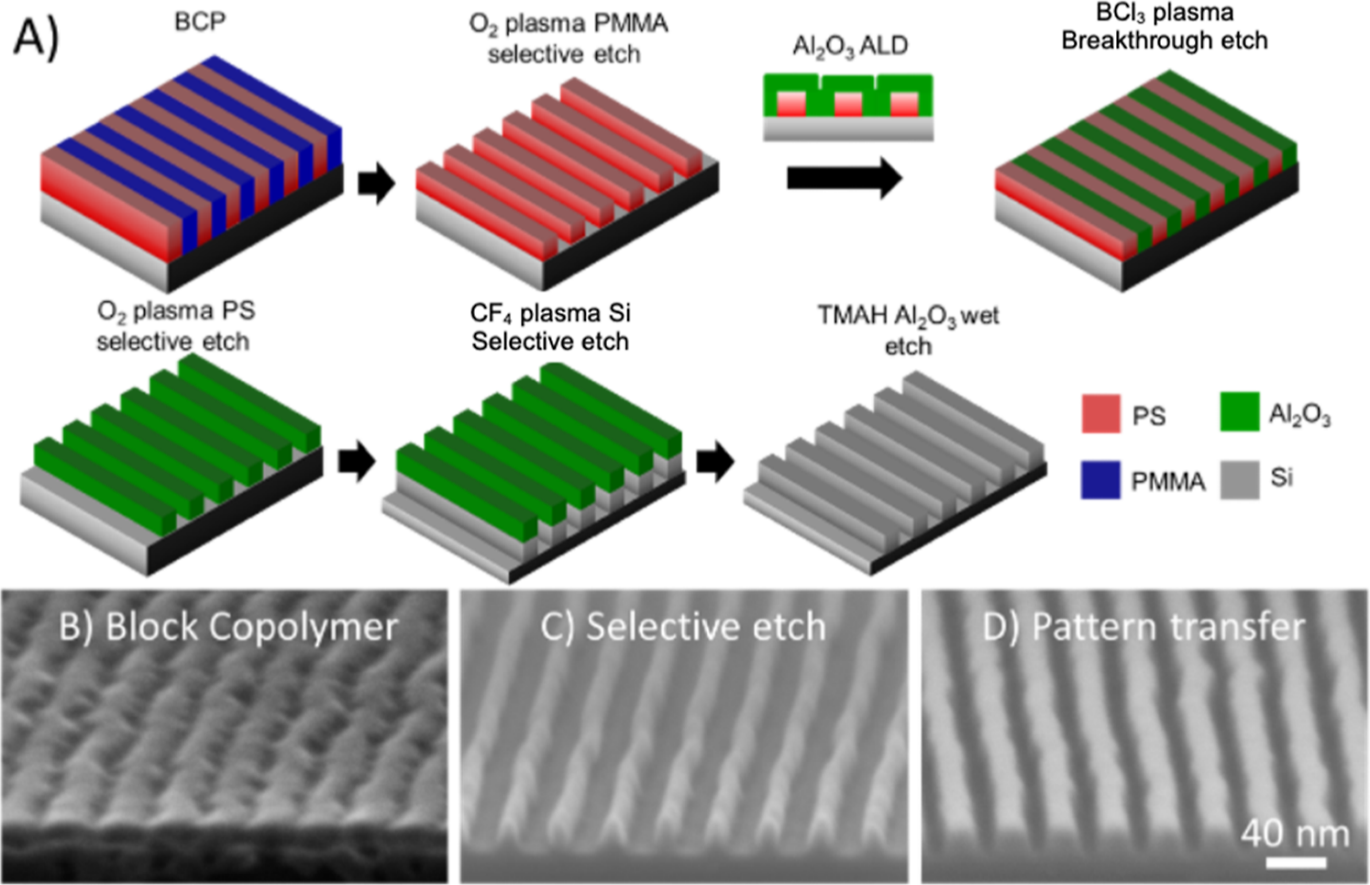
Pattern Transfer. (a) Workflow for the reverse tone “dry lift-off” pattern transfer. (b) Cross-section of the DSA of a thick film (1.25*L*_0_). (c) PS pattern after selective dry etching of the PMMA block. (d) Si pattern after pattern transfer via the “dry lift-off” protocol.

The ability to obtain defect-free DSA using films with a thickness greater than 1*L*_0_ also increases the latitude for pattern transfer due to a larger amount of material available for etching. Here, we demonstrate a reverse-tone “dry lift-off” protocol involving a series of dry etching steps, ALD, and hard mask removal (Figure 6). First, the PMMA domain is selectively etched (2:1) relative to PS using O_2_ plasma (Figure 6b,c). Al_2_O_3_ is conformally deposited using ALD, which fills the trenches forming a planarization layer. The ALD is done at 40 °C below the glass-transition temperature of PS to ensure that the structural integrity of the PS pattern is retained during deposition of the hard mask. The conformal Al_2_O_3_ fills the gaps and planarizes the PS pattern. BCl_3_ plasma etch is then used to break through the top Al_2_O_3_ layer, exposing the interdigitated PS and Al_2_O_3_ domains. The PS is subsequently removed using O_2_ plasma, creating a reversed tone Al_2_O_3_ hard mask. The exposed Si is then etched using CF_4_/CHF_3_/Ar plasma, and the Al_2_O_3_ hard mask is removed via TMAH wet etch (Figure 6d). This dry lift-off pattern transfer approach, while similar to recent planarization demonstrations,^29,30,44^ highlights the value of combining ALD with BCP lithography. The pattern transfer method can be easily scaled with decreasing feature sizes once a PS soft mask can be generated from the BCP pattern. We further hypothesize that many of the issues observed at the substrate during pattern transfer of BCP patterns from xPS guiding stripes, especially those with *W*_s_ = 1.5*L*_0_, will not be observed during pattern transfer of BCPs from S_OH_ guiding stripes due to the matched etch rates between PS and S_OH_ as opposed to the decreased etch rate of xPS as well as the uniformity in the cross-section of the S_OH_ guide stripes.^37,45^ As these patterns are scaled down toward smaller sub-10 nm dimensions, the cross-section uniformity of the remaining copolymer block pattern will become increasingly important for successful pattern transfer.

## Conclusions

In summary, we demonstrate a self-healing DSA workflow that improves the quality of the chemical contrast pattern by the sequential grafting of homopolymer brushes, with tolerance for simultaneous variations in the dimensions and chemical composition of the chemical contrast patterns. The effects of polymer chain interpenetration into the brush layer can be either mitigated or exploited by the appropriate choice of ink molecular weight. For instance, in the guiding patterns, we chose S_OH_ to comprise the longest chains on the brush layer so that the interpenetration of subsequent chains would not alter the chemical affinity or wetting properties at the top surface. On the other hand, the shortest molecules were selected for the random copolymer brushes on the background region such that subsequent S_OH_ and M_OH_ would stand taller above the SrM_OH_, masking the surface properties of the random brush and creating 1:1 guiding patterns matching the BCP dimensions. This occurs despite a limited number of chains being inserted due to steric hindrance from the existing brush monolayer. However, during the SRSA registration process, BCPs show patterns with higher LWR due to the local inhomogeneity in the distribution of the free polymer; this is not the case in films subsequently assembled on an SRSA-registered substrate. Finally, the SRSA substrate showed lower line placement and line edge roughness compared to those of DSA substrates for the same film thickness. The final BCP patterns were successfully transferred into the Si substrate using a reverse tone “dry lift-off” protocol. The DSA workflow presented here could serve as a pathway for routine implementation without the need for cutting edge tools, materials, and processing conditions. This potentially expands the adoption of DSA to a broader community that could benefit from self-assembled structures with a long range order. We also anticipate that a wide process window workflow that uses only brushes and no mats could facilitate scaling of DSA and pattern transfer below 10 nm; a regime where DSA could augment EUV lithography.

## Supporting Information Available

Additional characterization of chemical templates throughout the sequential brush process and roughness measurements (PDF)

## Symbol

*L*_0_: block copolymer pitch
*L*_s_: pitch of chemical guiding patterns
*t*: film thickness
*W*_S_: width of guiding stripe

## Greek Symbol

χ: Flory–Huggins interaction parameter
3σ: overall roughness

## Abbreviations

ALD: atomic layer depositon
BCP: block copolymer
DSA: directed self-assembly
EBL: electron beam lithography
EUV: extreme ultraviolet
LER: line edge roughness
LPR: line placement roughness
LWR: line width roughness
M: PMMA domain of BCP
M_OH_: PMMA-OH brush
MTP: molecular transfer printing
NMP: *N*-methyl-2-Pyrrolidone
PMMA: poly(methyl methacrylate)
PS: polystyrene
PSD: power spectral density
S: PS domain of BCP
S_OH_: PS-OH brush
SbM: polystyrene-*block*-poly(methyl methacrylate)
SrM_OH_: polystyrene-random-poly(methyl methacrylate)-OH brush
xPS: cross-linkable polystyrene
