# Supplementary material for: Sequential Brush Grafting for Chemically and Dimensionally Tolerant Directed Self-Assembly of Block Copolymers
Source: ACS Appl Mater Interfaces. 2022 Dec 19;15(1):2020–9. doi: 10.1021/acsami.2c16508 (PMC9837782; doi:10.1021/acsami.2c16508)
Supplement: Supplementary file 1 — am2c16508_si_001.pdf [file am2c16508_si_001.pdf]

Supporting Information for

Sequential Brush Grafting for Chemically and Dimensionally Tolerant Directed-Self Assembly of Block Copolymers

Boyce Chang[†§], Whitney Loo[‡§], Beihang Yu[†], Scott Dhuey[†], Lei Wan[±], Paul Nealey[‡⊥], Ricardo Ruiz[†,*]

[†] Molecular Foundry, Lawrence Berkeley National Lab, Berkeley, CA, USA 94720

[‡] Pritzker School of Molecular Engineering, University of Chicago, Chicago, IL, USA, 60637

[±]Western Digital, San Jose, CA, USA, 95119

[⊥]Argonne National Lab, Lemont, IL, USA 60439

[§] Equal contribution

*Corresponding Author: RR (rruiz@lbl.gov)

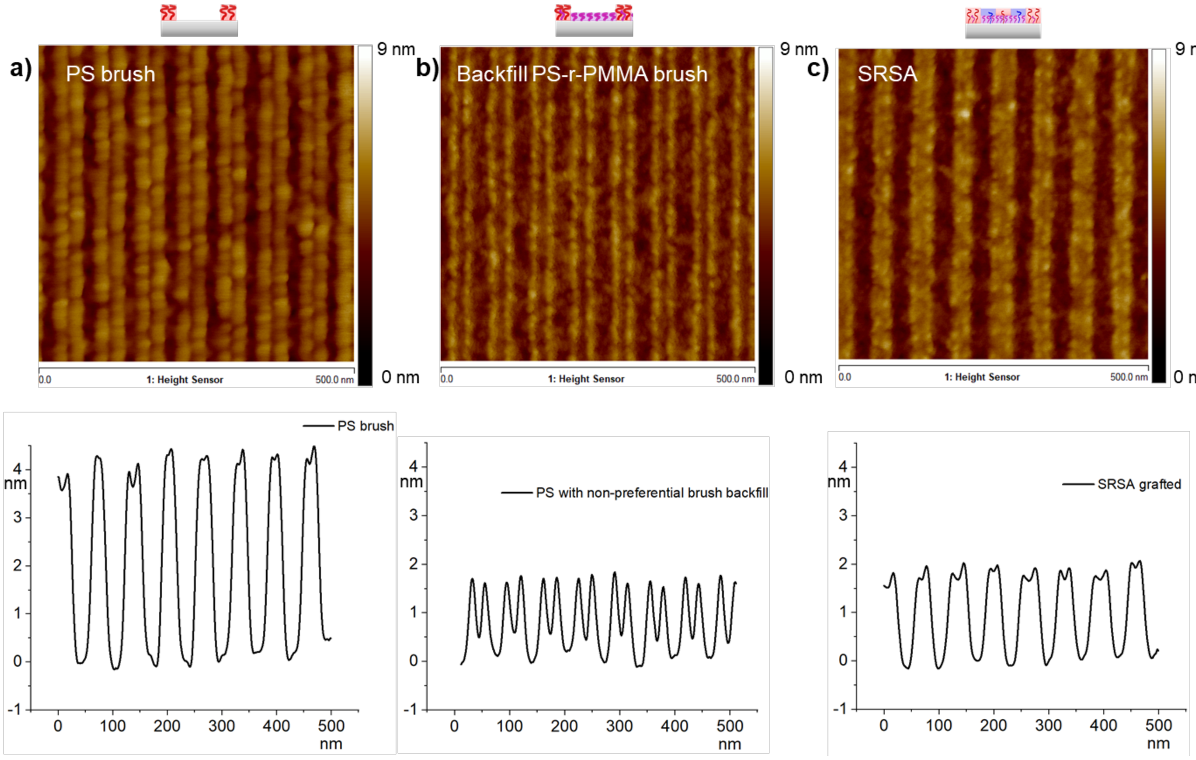

Figure S1: Topography of the chemical patterns at various steps of the workflow for a) S_{OH} chemical guiding pattern, b) S_{OH}+SrM_{OH} backfilled chemical guiding pattern and c) SRSA grafted chemical guiding pattern following rinsing of the SbM + inks film.

Roughness Measurement Methods

Roughness measurements were taken from square SEM images 2048 x 2048 pixels in size with a pixel size of 0.744 nm for a total image size of 1523 x 1523 nm. Line extraction was carried out by an image processing described elsewhere.[1] The definitions for line edge, width, and placement roughness as well as the correlation coefficients can be found in ref 2.

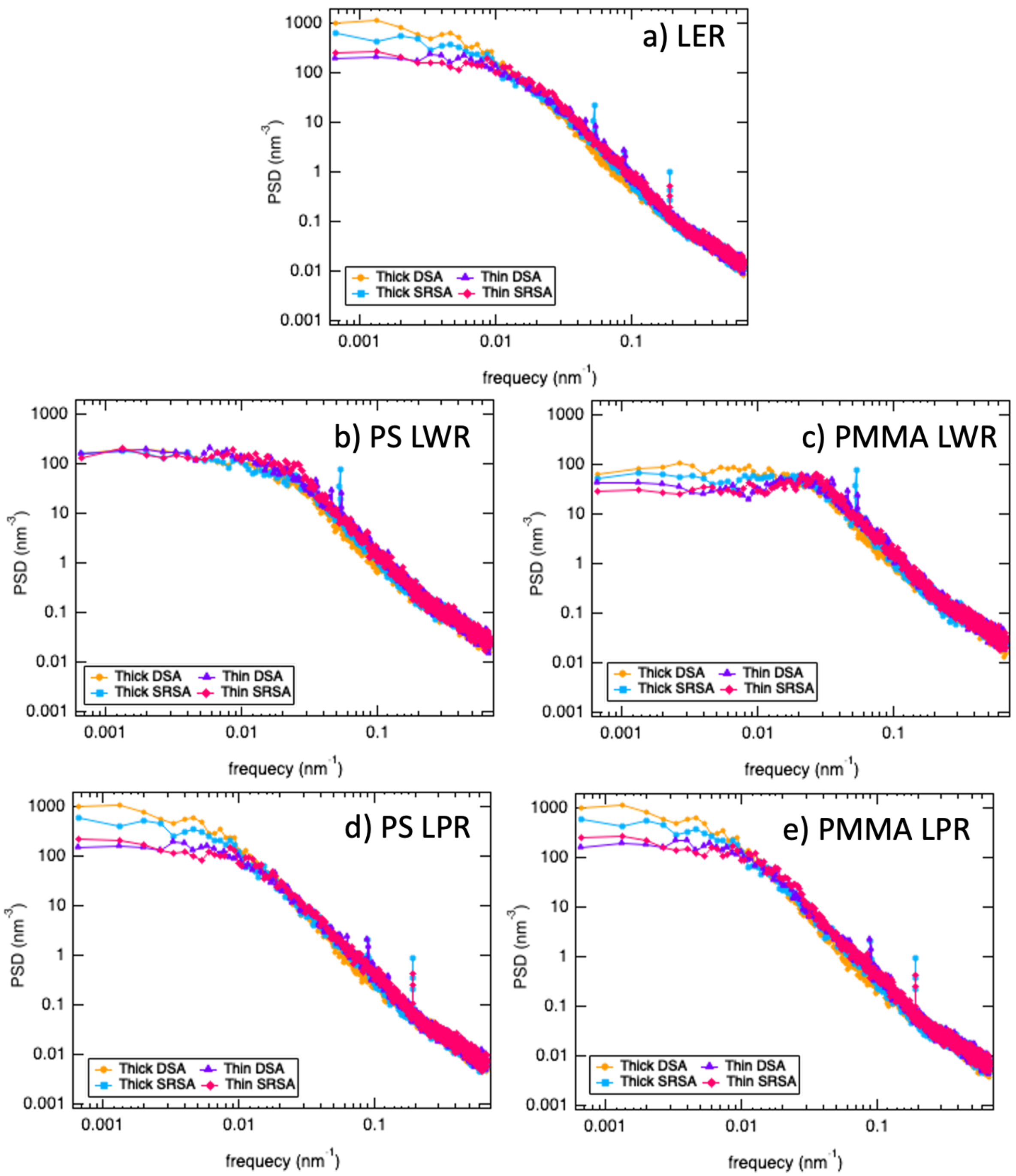

Figure S2: Power spectral densities plots for a) line edge roughness (LER), b) PS line width roughness (LWR), c) PMMA LWR, d) PS line placement roughness (LPR) and e) PMMA LPR for thick and thin DSA and SRSA patterns.

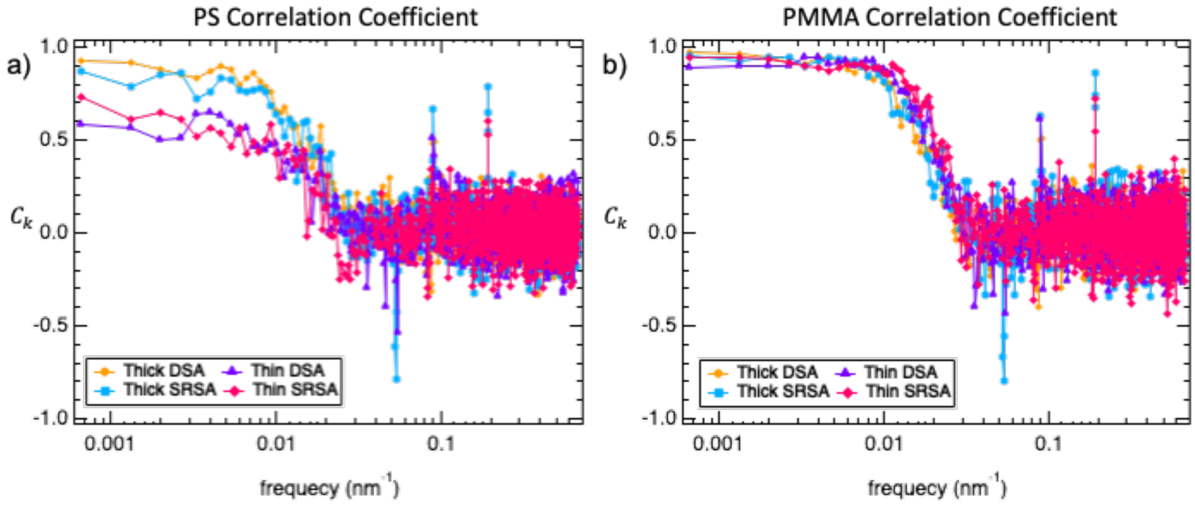

Figure S3: Frequency dependent correlation coefficients for a) PS and b) PMMA for thick and thin DSA and SRSA patterns.

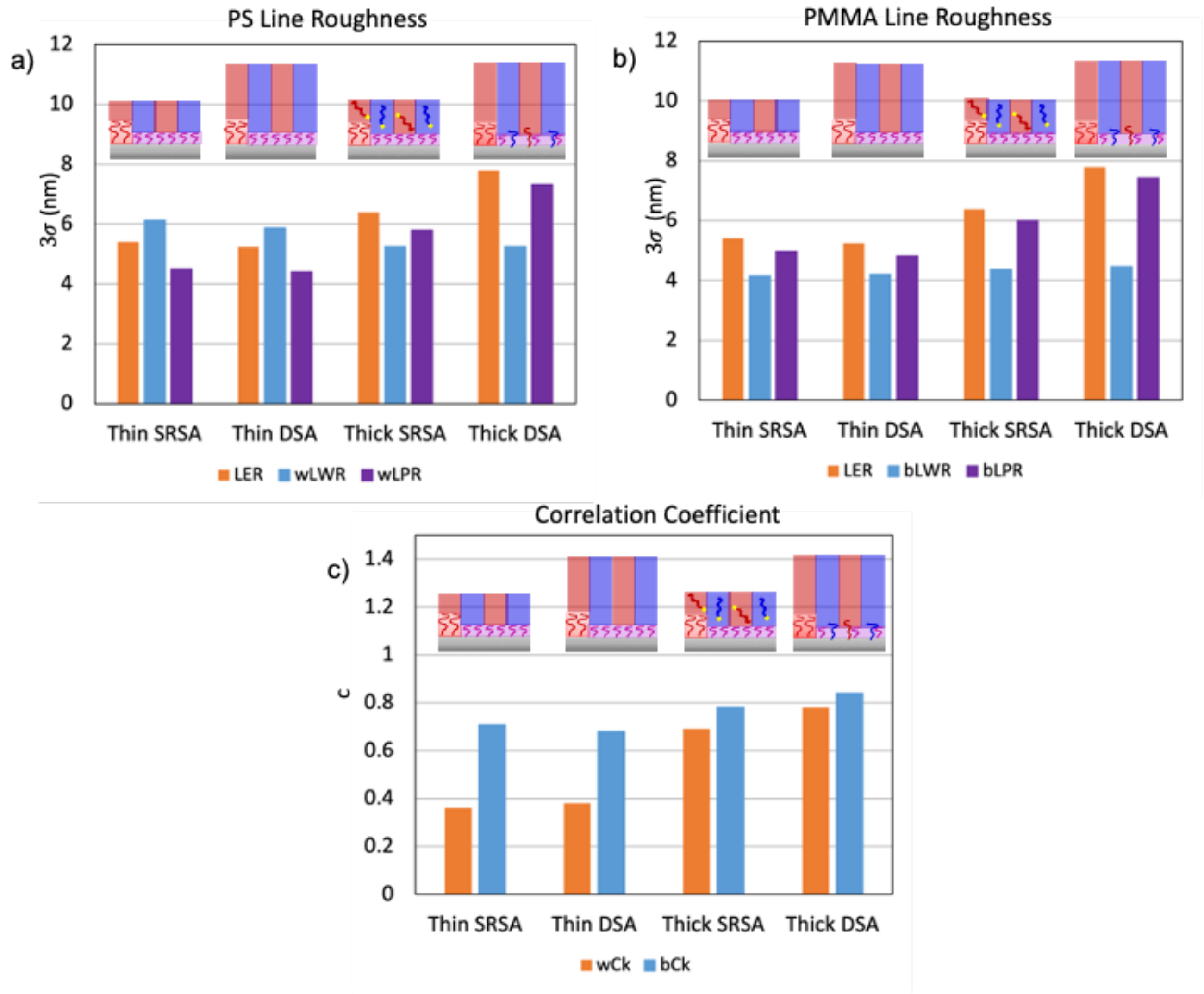

Figure S4: RMS roughness values, 3σ (nm), for a) PS line roughness, b) PMMA line roughness and c) correlation coefficients for thin and thick DSA and SRSA

References

(1) Lorusso, G. F.; Sutani, T.; Rutigliani, V.; Van Roey, F.; Moussa, A. Need for LWR Metrology Standardization: The Imec Roughness Protocol. *J. Micro/Nanolithography, MEMS, MOEMS* **2018**, *17* (04), 1. https://doi.org/10.1117/1.jmm.17.4.041009.

(2) Ruiz, R.; Wan, L.; Lopez, R.; Albrecht, T. R. Line Roughness in Lamellae-Forming Block Copolymer Films. *Macromolecules* **2017**, *50* (3), 1037–1046. https://doi.org/10.1021/acs.macromol.6b02399.
